# Machine Learning Based Classification of Resting-State fMRI Features Exemplified by Metabolic State (Hunger/Satiety)

**DOI:** 10.3389/fnhum.2019.00164

**Published:** 2019-05-28

**Authors:** Arkan Al-Zubaidi, Alfred Mertins, Marcus Heldmann, Kamila Jauch-Chara, Thomas F. Münte

**Affiliations:** ^1^Department of Neurology, University of Lübeck, Lübeck, Germany; ^2^Institute for Signal Processing, University of Lübeck, Lübeck, Germany; ^3^Institute of Psychology II, University of Lübeck, Lübeck, Germany; ^4^Department of Psychiatry and Psychotherapy, Kiel University - Christian-Albrechts, Kiel, Germany

**Keywords:** brain functional activity and connectivity, feature selection, resting-state fMRI, hunger, satiety, support vector machine

## Abstract

**Objective:**

Resting-state functional magnetic resonance imaging (rs-fMRI) has become an essential measure to investigate the human brain’s spontaneous activity and intrinsic functional connectivity. Several studies including our own previous work have shown that the brain controls the regulation of energy expenditure and food intake behavior. Accordingly, we expected different metabolic states to influence connectivity and activity patterns in neuronal networks.

**Methods:**

The influence of hunger and satiety on rs-fMRI was investigated using three connectivity models (local connectivity, global connectivity and amplitude rs-fMRI signals). After extracting the connectivity parameters of 90 brain regions for each model, we used sequential forward floating selection strategy in conjunction with a linear support vector machine classifier and permutation tests to reveal which connectivity model differentiates best between metabolic states (hunger vs. satiety).

**Results:**

We found that the amplitude of rs-fMRI signals is slightly more precise than local and global connectivity models in order to detect resting brain changes during hunger and satiety with a classification accuracy of 81%.

**Conclusion:**

The amplitude of rs-fMRI signals serves as a suitable basis for machine learning based classification of brain activity. This opens up the possibility to apply this combination of algorithms to similar research questions, such as the characterization of brain states (e.g., sleep stages) or disease conditions (e.g., Alzheimer’s disease, minimal cognitive impairment).

## Highlights

- We compare fALFF, DC, and ReHo for classifying human metabolic states within an rs-fMRI scan based on SVM.
- We combine an rs-fMRI based voxel-wise frequency-domain approach with sequential forward floating selection method to identify brain areas modulated as a function of hunger/satiety.
- It turns out that fALFF is a reliable and stable index of spontaneous brain activity.

## Introduction

Resting-state functional magnetic resonance imaging (rs-fMRI) has been increasingly applied to study activity and connectivity of the resting brain and involves the recording of the blood-oxygen-level-dependent (BOLD) signal without imposing a task (Biswal et al., 2010; X.-N. Zuo and Xing, 2014). This approach can be used to assess intrinsic and spontaneous brain activity. Analysis techniques of rs-fMRI have evolved rapidly over the past few years and are based on correlation methods (Bullmore and Sporns, 2009; Sporns, 2011; Wee et al., 2012; Jie et al., 2014), partial correlation (Salvador et al., 2005; Marrelec et al., 2006, 2007), graph theory based analysis (Chen and Herskovits, 2007; Bullmore and Sporns, 2009) and sparse representation methods (Lee et al., 2011; Wee et al., 2014), among others. Because of its simplicity (short scan time, no stimulation equipment needed, no task requirements), the rs-fMRI method has become particularly popular for the characterization of clinical conditions, for example pinpointing to differences between healthy participants and patients with Parkinson’s disease (Göttlich et al., 2013; Tahmasian et al., 2015), Alzheimer’s disease (AD) (Sheline and Raichle, 2013; Dennis and Thompson, 2014), bilateral vestibular failure (Göttlich et al., 2014a), schizophrenia (Alderson-Day et al., 2016; Hu et al., 2017), and obsessive-compulsive disorder (Göttlich et al., 2014b; Gürsel et al., 2018), to name but a few targeted neuropsychiatric conditions.

In neuroimaging, machine learning classifier (MLC) methods are applied to fMRI data to detect model-free brain activity and to use these brain activity patterns to differentiate between groups or conditions (Cox and Savoy, 2003; Pereira et al., 2009). The application of MLC to fMRI data is often referred to as multi-voxel (i.e., analyzing more than one voxel at once) pattern analysis (MVPA). MVPA is a helpful tool to investigate how a pattern of brain activity is related to different cognitive states (Haynes and Rees, 2006; Norman et al., 2006; Mahmoudi et al., 2012). The process of applying the MVPA approach to fMRI data can be broken down into three stages (Pereira et al., 2009; Poldrack et al., 2011). First, feature extraction, which converts the BOLD fMRI signals to the relevant variables, i.e., features, which will be used to train and to test the classifier. Second, feature selection, which determines features that have to be included in the classifier analysis in order to improve the classification. Third, cross-validation methods that divide the data into training and testing samples and determine the accuracy of the classifier in generalization to new data.

Support vector machine (SVM; Cortes and Vapnik, 1995; Vapnik, 2013) is a powerful method available to perform MVPA. In contrast to alternative MVPA methods (such as linear discriminant analyses), SVM provides better prediction accuracy, having the advantage of being relatively insensitive to the sample size of the training dataset (O’Toole et al., 2007; Mountrakis et al., 2011). Furthermore, SVM has additional advantages regarding efficiency, simplicity, robustness and is less susceptible to noise (Mountrakis et al., 2011; Meier et al., 2012).

The application of SVM to fMRI data at group level has several advantages over traditional univariate (i.e., individual) voxel-based methods, like the general linear model (GLM). For instance, SVM allows to identify voxels or brain regions of interest that are informative for classifying groups by accumulating the information in an efficient way across many spatial locations. While in GLM analysis, these voxels or brain regions could appear statistically insignificant, although they might carry some information about differences between states or groups (Haynes and Rees, 2006; Norman et al., 2006). Thus, SVM provides insight into the defining differences between the two states or groups (Cox and Savoy, 2003; Orru et al., 2012).

In many cases, fMRI data have a small number of samples and a large number of variables or features. This often leads to overfitting in classification, which in turn leads to deceptive diagnostic results and poor generalization performance (Pereira et al., 2009; Wang, 2009). To avoid the danger of overfitting, most of the MVPA-based fMRI studies applied both methodologies, feature-selection algorithms to remove redundant information and MLC methods that are less sensitive to a high dimensionality, such as linear SVM. Finally, cross-validation analyses are performed to evaluate the classification accuracy and generalizability for unseen data (Cox and Savoy, 2003; Mitchell et al., 2004; Kamitani and Tong, 2005; Zhang et al., 2005).

Rs-fMRI yields data comprise multiple data points per subject and/or condition, among other things, raising the question of whether it might be possible to distinguish between different conditions (e.g., disease present or not) using classification algorithms from the realm of machine learning. Indeed, several recent publications have tackled this question. For example, Abós et al. (2017) obtained functional connectomes from the rs-fMRI in healthy controls (HC) and 70 Parkinson’s disease patients [of which one third had a mild cognitive impairment (MCI)]. Using a SVM trained on features selected through randomized logistic regression with leave-one-out cross-validation (LOOCV), they could separate patients with MCI from those not having MCI with an accuracy of about 83% in the training sample. In a smaller validation sample of 25 Parkinson patients (8 MCI), classification accuracy with regard to MCI was 80% using the features found in the training sample (Abós et al., 2017). This suggests that SVM classification based on metrics obtained from rs-fMRI can indeed yield meaningful results. Likewise, applying a graph theoretical approach to rs-fMRI to characterize functional connectivity in patients with MCI, AD and age-matched HC (total sample *n* = 168), followed by SVM based classification, Khazaee et al. (2015a) were able to accurately classify the subjects into three groups (HC, MCI, and AD) with 88.4% accuracy. The same research group (Hojjati et al., 2017) tried to distinguish patients with MCI who later converted to an AD from MCI patients who did not. Again a SVM, using features derived from local and global graph measures, was used. This approach yielded a specificity of 91.4% and sensitivity of 83.2% regarding the conversion to the AD. Bi et al. (2018) attempted to classify patients with autism spectrum disorder (ASD) from HC using random SVM cluster and reported classification accuracy based on the optimal feature to be 96%. These are just a few examples illustrating that rs-fMRI derived features can be used for classification of conditions using machine learning algorithms. What it is less clear, however, is which method of rs-fMRI analysis delivering the most discriminating features might be best in distinguishing different metabolic states (hunger vs. satiety).

In the present investigation, we therefore sought to compare the accuracies of three different connectivity parameters or features (the predictor variables used for classification) extracted from rs-fMRI fluctuations. These features assess local and global functional connectivity as well as changes in the brain activity as indicated by the amplitude of the BOLD signal, i.e., regional homogeneity (ReHo), degree of centrality (DC), and fractional amplitude of low-frequency fluctuations (fALFF), respectively. Briefly, ReHo characterizes the local connectivity of a brain voxel to its nearest neighboring brain voxels (Zang et al., 2004; Jiang and Zuo, 2015) by determining the coherence among spontaneous BOLD signals that might reflect spontaneous neuronal activity (Sato et al., 2012). ReHo has been applied to widely differing neuropsychiatric conditions (Cao et al., 2006; Liu et al., 2006; He et al., 2007; Wu et al., 2009; Paakki et al., 2010). DC is derived from graph theory based analysis and describes the global connectivity (global connectedness) of a given voxel with the voxels in the entire brain, by computing the number of connections above a certain threshold (Buckner et al., 2009; Bullmore and Sporns, 2009; Zuo et al., 2012). Again, DC has seen widespread application in neuropsychiatric conditions (Buckner et al., 2009; Beucke et al., 2013; Di Martino et al., 2013; Göttlich et al., 2013; Hou et al., 2014). Finally, to quantify spontaneous local brain activity, the amplitude of the BOLD signals has been used. This can be assessed by the amplitude of low-frequency fluctuations (ALFF) and its derivative fALFF (Zang et al., 2007; Zou et al., 2008). While ALFF describes the local spontaneous brain activity across the whole brain, by assessing the amplitude in a given voxel or brain area in the low-frequency range (0.01–0.08 Hz), fALFF is a normalized derivation of ALFF representing the ratio of low-frequency range amplitudes (0.01–0.08 Hz) relative to the entire frequency range (e.g., 0–0.25 if TR=2 s) amplitudes. Both ALFF and fALFF have high temporal stability (Küblböck et al., 2014) and test-retest reliability (X. N. Zuo and Xing, 2014). In contrast to ALFF, fALFF has been reported to have higher specificity in detecting local spontaneous brain activities, especially in gray matter (Zou et al., 2008; Zuo et al., 2010). Moreover, fALFF is recommended to be used instead of ALFF (X.-N. Zuo and Xing, 2014), since it is more robust against non-specific signal components, such as physiological noise (Zuo et al., 2010). In the present study, we performed fALFF on rs-fMRI data to describe the local spontaneous brain activities.

The aim of feature selection algorithms is to reduce the dimensionality of feature space and computation time, as well as to enhance the accuracy of optimization methods by ignoring redundant, irrelevant or noisy features (Tang et al., 2014; Jović et al., 2015). In general, the feature selection algorithms are classified in two categories, according to the type of objective functions that one chooses to work with: filter methods and wrapper methods (Pereira et al., 2009; Mwangi et al., 2014). Filter methods select the feature subsets based on statistical properties (such as interclass distance, mutual information, entropy or statistical independence) of the features to filter out poorly informative ones without employing any classification algorithm. In contrast, wrapper methods rate the feature subsets based on their predictive accuracy to improve the performance of classification when applying a particular classifier (such as SVM or the k-nearest neighbor). Filter methods are advantageous because they perform quickly, afford a more general solution and tend to select large feature subsets. Wrapper methods are expensive because they need more time to train the classifier of each subject many times (i.e., cross-validation), but often do not deteriorate from the problem of overfitting (Burrell et al., 2007) and provide more accurate results comparable to filter methods (Guyon and Elisseeff, 2003; Maldonado and Weber, 2009).

There are several strategies to apply wrapper methods (Mwangi et al., 2014). For instance, sequential forward selection (SFS) and sequential forward floating selection (SFFS) are easy to execute and are assumed to provide useful results. Although the SFFS strategy requires massive computational resources, it performs better and is more effective for solving small- and medium-scale problems than simpler strategies like SFS (Kudo and Sklansky, 2000). However, the SFS strategy reduces the computational costs for the feature subset selection. Accordingly, Burrell et al. (2007) concluded that SFS was a reasonable alternative to select a small subset of features for fMRI data. In this work, we compared between SFS and SFFS strategies for creating feature subsets to distinguish different metabolic states.

**FIGURE 1:**
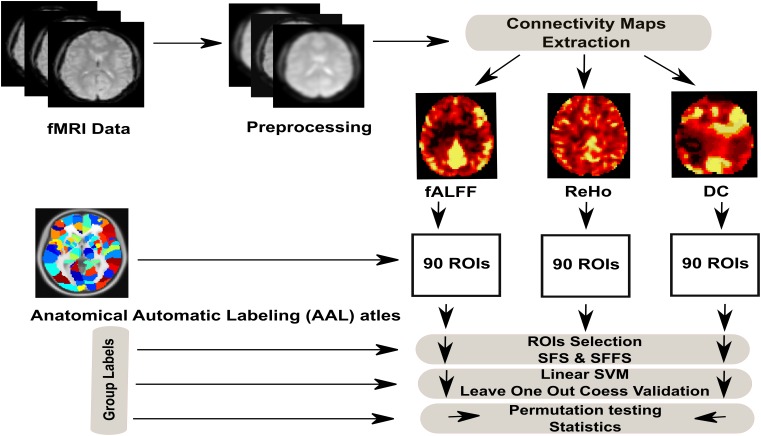
Full analysis procedure of hunger classification based on rs-fMRI data.

The emphasis of the present work is on the ability to classify the metabolic states (hunger vs. satiety) by the MVPA approach. Therefore, we first estimate and compare the prediction accuracy of classification (hunger vs. satiety) based on different features of rs-fMRI data (ReHo, DC and fALFF). Second, we identify brain regions containing discriminating information between different metabolic states. To this end, we apply supporting linear SVM as a classifier and two feature selection strategies (SFS and SFFS) to identify those brain regions that most efficiently differentiate between hungry and satiated states based on rs-fMRI data. Finally, we employ a cross-validation scheme and permutation tests to validate the reliability of classifier and significance testing, respectively (see Figure 1 for classification procedure).

## Materials and Methods

### Experimental Design

We investigated 24 healthy male volunteers aged 20 to 30 years (mean ± SEM: 24.5 ± 0.6 years) with a body mass index (BMI) within the normal weight range of 20–25 kg/m^2^ (mean ± SEM in kg/m^2^: 23.4 ± 0.3), recruited from the University and the local population of Lübeck, Germany.

Each subject was investigated in two sessions (i.e., two metabolic states), once under fasting (36 h fasting) and once under standardized eating conditions (five meals throughout 36 h). The order of the two sessions was randomized across participants, with a break of at least 1 week between sessions.

In the hunger condition, subjects fasted (no food or beverages, except water) from 11 pm the night before the examination started until the end of the rs-fMRI recording. In the satiety condition, three standardized meals per day were provided. Standardized meals were served according to the recommendations of the clinical diabetes counseling department at the University Medical Campus Schleswig-Holstein (UMCSH): Breakfast (25% protein, 50% carbohydrate and 25% fat), lunch (20% protein, 63% carbohydrate and 17% fat) and dinner (22% protein, 60% carbohydrate and 18% fat) were provided at 9 am, 12 pm and 7 pm, respectively. In both sessions, subjects arrived at the sleep lab at 8 am. The first blood sample for defining the blood glucose levels was taken at 8:45 am. More details on the exact timing for obtaining blood samples can be found in our previous work (please see the experimental design and Figure 1 in Al-Zubaidi et al., 2018). All subjects stayed and slept overnight in the laboratory at the UMCSH. On the next morning, blood samples were drawn at 8:45 am and at fixed time points throughout noon until the MRI was performed at 1 pm for the rs-fMRI recording (duration 6 min). Subjects were instructed to lie still inside the scanner having the eyes closed and trying to avoid any particular cognitive activity. For each condition, subjects rated hunger feelings 20 min before the MRI sessions started. This was done by using a visual analog scale from 0 (not hungry at all) to 9 (very hungry).

**FIGURE 2:**
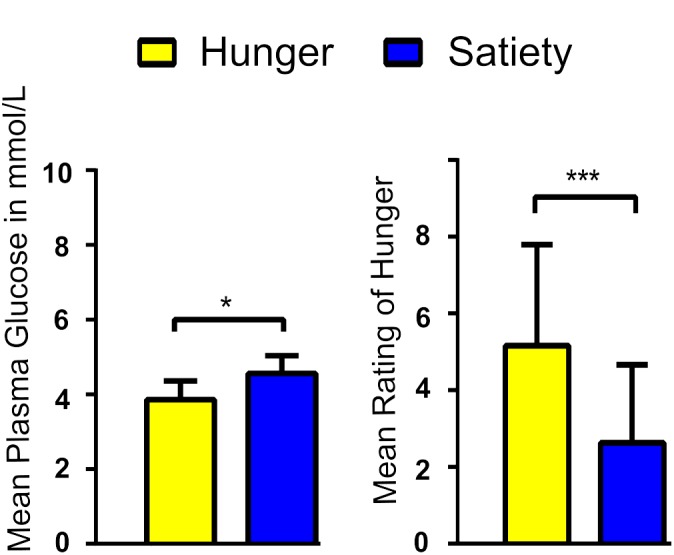
Average blood glucose levels **(A)** and rating of hunger feeling **(B)** per experimental condition. ^∗^ and ^∗∗∗^ represent the significant differences between conditions, at a threshold of *p* < 0.01 and *p* < 0.0001, respectively.

Blood sugar levels were lower while feelings of hunger were more intense during hunger compared to satiety before the rs-fMRI scans (Figure 2). These findings confirm the success of our fasting treatment. Results of blood sugar levels and hunger ratings can be found in Al-Zubaidi et al. (2018).

### Image Acquisition

We used a 3-T Philips Achieva scanner (Philips Medical Systems, Best, Netherlands) and a standard eight-channel phased array head coil to record structural and functional images. Anatomical scans consisting of 180 sagittal slices were acquired by applying a T1-weighted 3D turbo gradient-echo sequence with SENSE (image matrix 240 × 240; voxel dimensions 1 × 1 × 1 mm; field of view 240 × 240 mm^2^; 1 mm slice thickness; 9° flip angle). For the rs-fMRI recording (duration 6 min), subjects were instructed to lie still inside the scanner with their eyes closed and to not engage in any particular cognitive activity. Further, 178 whole-brain fMRI volumes were acquired in an interleaved fashion with a T2^∗^-weighted single-shot gradient-echo EPI sequence (TR = 2000 ms; TE = 28 ms; voxel dimensions 3 × 3 × 3 mm; field of view 192 × 192 mm^2^; 80° flip angle; 40 slices).

### Preprocessing

Part of the preprocessing of the functional images was carried out using FSLv5.0^1^ to implement independent component analysis (ICA)–based strategy for automatic removal of motion artifacts (ICA-AROMA) for head motion correction (Pruim et al., 2015a). It has been reported that ICA-AROMA improves the specificity (i.e., the signals of interest) and sensitivity (i.e., motion-related noise) of rs-fMRI activation and connectivity analyses (Pruim et al., 2015a). To improve inter-subject alignment (Klein et al., 2009; McLaren et al., 2010), the spatial preprocessing of the data was performed with the statistical parametric mapping 12b (SPM12b).

The rs-fMRI images were preprocessed as follows: (i) we discarded the first eight functional volumes from each participant’s two runs to allow steady-state tissue magnetization; (ii) we manually reoriented all functional volumes to the anterior commissure; (iii) we implemented head movement correction on data by realigning all volumes to the middle time-point volume using MCFLIRT (Jenkinson et al., 2002); (iv) we applied ICA-AROMA to the data in order to identify and remove motion-related components using four spatial and temporal features (Pruim et al., 2015b). Briefly, functional images were submitted to the MELODIC toolbox for running a probabilistic independent component analysis (PICA) with automatic estimation of the number of hidden independent components (i.e., source signals) to find a set of components for each participant per recording individually (Beckmann and Smith, 2004). A predetermined classifier was then applied on independent components to represent the motion-related artifact components, which were identified at least by assessing each component to one of the following criteria: (1) maximum correlation with realignment parameters, (2) high-frequency temporal content >35%, (3) spatial content in edge voxels and cerebrospinal fluid (CSF) >10%. Finally, we removed the motion-relevant components from rs-fMRI data that had been realigned by using a linear regression approach. The structural and denoised functional images were further preprocessed with data processing assistant for resting-state fMRI (DPARSF) toolbox as follows (Yan, 2010): (v) we co-registered the T1 structural image to the mean functional image; (vi) we ran a segmentation protocol to distinguish gray matter, white matter and CSF; (vii) we applied bias correction and spatial normalization of the T1 structural image and adjusted them to the MNI template using DARTEL algorithm (Ashburner and Friston, 2005); (viii) we performed nuisance regression (including white matter and CSF signals) to reduce the impact of undefined physiological effects on rs-fMRI signals (Liu, 2016); (ix) we spatially normalized functional images and gray matter images to the MNI-template using the normalization parameters estimated by DARTEL algorithm with a 3 mm isotropic voxel size; (x) we performed spatial smoothing with a 6 mm full width at half maximum (FWHM) Gaussian kernel. Rs-fMRI signals were smoothed after calculating ReHo (Zang et al., 2004) not before (see ReHo paragraph); (xi) we applied temporal band-pass filtering (0.01–0.08 Hz) on the rs-fMRI signal to reduce the effect of low-frequency drift, such as respiratory, and high-frequency noise, such as heart activity. As suggested by Zou et al. (2008) no such band-pass filter was used when computing the fALFF (see fALFF paragraph); (xii) finally, we masked all functional images with a gray matter mask. The gray matter mask was calculated by averaging the grey matter images of all subjects. To generate the binary mask, we defined the common voxels between the average gray matter image and the gray matter template (without cerebellar lobules) derived from the Automated-anatomical-labeling (AAL) atlas (Tzourio-Mazoyer et al., 2002) by using the xjView toolbox^2^. In some subjects we were not able to measure the whole cerebellum. Therefore, the cerebellar cortex was excluded from the gray matter mask and regions of interest (ROIs).

### Feature Extraction From rs-fMRI Data

Feature extraction is used to reduce the dimension of the original data space to a new feature space. This new feature space helps to minimize the training time taken by the classifier (Chen et al., 2010). To encompass different aspects of rs-fMRI fluctuations, we extracted and compared three of the most common features according to their accuracy to select those brain regions that best distinguish different metabolic states by using linear SVM together with feature selection strategies. In the beginning, we submitted the preprocessed data to the DPARSF toolbox (Yan, 2010) and extracted three features from each subject per section as described in the following paragraphs. Then, those features were analyzed as explained in Section of Feature Selection.

First, local connectivity of brain regions was described using ReHo (for regional homogeneity), which is a measure of the temporal homogeneity among brain voxels and the neighboring brain voxels within the low-frequency range of rs-fMRI signals (Zang et al., 2004). The rationale behind ReHo is based on the assumption that the BOLD signal has characteristics that depend on neuronal activities, and therefore, the time series of neighboring voxels in a functional brain area will be highly similar or synchronized when that area supports specific goals or representations (Jiang and Zuo, 2015). The ReHo index for a particular voxel is calculated by using Kendall’s coefficient concordance (KCC) approach. In this study, the KCC was calculated using the following formula (Zang et al., 2004):

$$\mathrm{KCC}=\frac{\sum_{i=1}^{n}{\left(R_{i}\right)}^{2}-n{\left(\bar{R}\right)}^{2}}{\frac{1}{12}K^{2}(n^{3}-n)}$$

where KCC is the ranging coefficient, from 0 to 1 (no to maximal coherence), of a given voxel in relation to its nearest neighbors, *R*_i_ represents the rank sum of *i* th time point as $R_{i}=\sum_{j=1}^{k}r_{\mathrm{ij}}$ and *r*_ij_ is the rank of the *i* th time point of the *j* th voxel. $\bar{R}$ refers to the average of the *R*_i_ and *n* represents the length of the time series (here *n* = 170 time points). *K* is the number of voxels within the targeted clusters (here *K* = 27, the given voxel (which is the center voxel) plus its 26 immediate neighbors). The KCC value was then put to the center voxel of the respective cluster. From the KCC of all voxels, the so-called ReHo map can be constructed. Thus, an individual ReHo map was computed for each subject per session.

Second, DC (for degree centrality) is used to investigate the global connectivity of brain regions, which is defined as the number of connections of one voxel in the brain to whole brain voxels (Buckner et al., 2009). This measure depends on graph theoretical approaches. The individual DC map was generated by correlating the time course of each voxel in the brain with all other voxels in the brain and calculating the number of connections above a definite threshold (Buckner et al., 2009; Al-Zubaidi et al., 2018). The temporal connection between two voxels was defined by applying Pearson’s correlation coefficient (*r*) approach. Then, the individual correlation coefficients were used to generate a correlation matrix = $\left[\begin{array}{ccc} r_{11} & ... & r_{1j}\\ \vdots & \ddots & \vdots \\ r_{i1} & \cdots & r_{\mathrm{ij}} \end{array}\right]$, 1 = *i*,*j* = *N*, where *N* is the number of voxels within the whole-brain mask and *r*_ij_ is the temporal Pearson’s correlation of time series between *i* th and *j* th voxels measuring the similarity between two voxels. The correlation matrix was thresholded at 0.25 to build a binary undirected and unweighted network matrix *d*_ij_ as follows

$$d_{ij}=\{\begin{array}{cc} 0, & r_{ij}<0.25\\ 1, & r_{ij}\geq 0.25 \end{array}$$

The binary connectivity matrix *d*_ij_ was used to define the degree centrality of voxel *D*_i_ by the following

$$D_{i}=\sum_{j=1}^{N}d_{ij}$$

Third, fALFF was employed to provide information on the magnitude of the BOLD signals, which reflects the neural activity of each brain voxel or region within a network of interest (Fujino et al., 2017; Al-Zubaidi et al., 2018). It refers to the ratio of rs-fMRI signal fluctuation in the low-frequency range proportional to the entire frequency range (Zou et al., 2008). DPARSF toolbox has a built-in fast Fourier transform (FFT) to convert time series for each voxel to the frequency domain and compute the power spectrum. This procedure estimates the amplitude of each frequency as the square root of the power spectrum. The total amplitude of the low-frequency range (0.01–0.08 Hz) was divided by that of the full frequency range 0–0.25 Hz.

### Feature Selection for Hunger/Satiety Status Classification

After generating ReHo, DC and fALFF maps from rs-fMRI data for each subject per condition, we used the AAL atlas to define the ROIs. The AAL atlas is a well-established anatomical parcellation of the brain into 45 ROIs per hemisphere when excluding the cerebellar lobules. Mean values of ReHo, DC and fALFF were calculated for each ROI and used to create a feature (region) vector, i.e., *R*[1, ..., 90], with 90 dimensions for each map. Those features are listed in Table 1.

**Table 1:** List of the anatomical regions (AAL atlas) of interest and their labels in the region vector.

Label	Anatomical	Label	Anatomical	Label	Anatomical
1	L. Amygdala	31	R. Sup. Frontal Med.	61	L. Sup. Parietal Gyrus
2	R. Amygdala	32	L. Sup. Frontal Orbital	62	R. Sup. Parietal Gyrus
3	L. Angular Gyrus	33	R. Sup. Frontal Orbital	63	L. Postcentral Gyrus
4	R. Angular Gyrus	34	R. Superior Frontal	64	R. Postcentral Gyrus
5	L. Calcarine Fissure	35	L. Fusiform Gyrus	65	L. Precentral Gyrus
6	R. Calcarine Fissure	36	R. Fusiform Gyrus	66	R. Precentral Gyrus
7	L. Caudate Nucleus	37	L. Heschl Gyrus	67	L. Precuneus
8	R. Caudate Nucleus	38	R. Heschl Gyrus	68	R. Precuneus
9	L. Ant. Cingulate Cort.	39	L. Hippocampus	69	L. Putamen
10	R. Ant. Cingulate Cort.	40	R. Hippocampus	70	R. Putamen
11	L. Mid. Cingulate Cort.	41	L. Insula	71	L. Rectus gyrus
12	R. Mid. Cingulate Cort.	42	R. Insula	72	R. Rectus gyrus
13	L. Pos. Cingulate Cort.	43	L. Lingual Gyrus	73	L. Rolandic Operculum
14	R. Pos. Cingulate Cort.	44	R. Lingual Gyrus	74	R. Rolandic Operculum
15	L. Cuneus	45	L. Inf. Occipital Gyrus	75	L. Supplementary Motor Area
16	R. Cuneus	46	R. Inf. Occipital Gyrus	76	R. Supplementary Motor Area
17	L. Inf. Frontal Oper.	47	L. Mid. Occipital Gyrus	77	L. Supramarginal Gyrus
18	R. Inf. Frontal Oper.	48	R. Mid. Occipital Gyrus	78	R. Supramarginal Gyrus
19	L. Inf. Frontal Orbital	49	L. Sup. Occipital Gyrus	79	L. Inf. Temporal Gyrus
20	R. Inf. Frontal Orbital	50	R. Sup. Occipital Gyrus	80	R. Inf. Temporal Gyrus
21	L. Inf. Frontal Triang.	51	L. Olfactory Cortex	81	L. Mid. Temporal Gyrus
22	R. Inf. Frontal Triang.	52	R. Olfactory Cortex	82	R. Mid. Temporal Gyrus
23	L. Med. Frontal Orbital	53	L. Pallidum	83	L. Mid. Temporal Pole Gyrus
24	R. Med. Frontal Orbital	54	R. Pallidum	84	R. Mid. Temporal Pole Gyrus
25	L. Frontal Middle	55	L. Paracentral Lobule	85	L. Sup. Temporal Pole Gyrus
26	L. Frontal Mid. Orbital	56	R. Paracentral Lobule	86	R. Sup. Temporal Pole Gyrus
27	R. Mid Frontal Orbital	57	L. Parahippocampal	87	L. Sup. Temporal Gyrus
28	R. Middle Frontal	58	R. Parahippocampal	88	R. Sup. Temporal Gyrus
29	L. Superior Frontal	59	L. Inf. Parietal Gyrus	89	L. Thalamus
30	L. Frontal Sup. Med.	60	R. Inf. Parietal Gyrus	90	R. Thalamus


*AAL, automated-anatomical-labeling; Ant, anterior; Cort, cortex; Inf, inferior; L, left; Med, ledial; Mid, middle; Oper, opercular; Pos, posterior; R, right; Sup, superior; Triang, triangular.*

In the classical classification problem, the goal of feature selection is to automatically search and select the best feature subset for the classification purpose. Here, we applied sequential feature selection algorithms to select the optimal feature subset (region subset) that best captured differences between hunger and satiety. This type of selection algorithm contains two components. The first element is a sequential search strategy to select and establish the best future subset, which evaluates additional features by a criterion function. In this study we used two strategies, namely SFS and SFFS. The SFS procedure starts by identifying the first feature with the highest classification rate and feeds it to a new empty candidate set. Other features are selected sequentially by adding a local feature to the first feature or the last subset of features in the candidate set, and testing a new feature combination until the highest classification rate (objective function) is achieved. The processing continues until further features do not enhance the objective function. However, the SFS algorithm is suboptimal and suffers from the “nesting effect” (Pudil et al., 1994), while SFFS offers the flexibility to discard features that were earlier selected and to re-evaluate features that had been discarded previously. This theoretical advantage notwithstanding, Burrell et al. (2007) showed that the computationally less demanding SFS could provide a reasonable alternative to SFFS to select features for discriminating between epileptic and non-epileptic activity of epileptic patients, indicating that both strategies had similar difficulties to separate patterns of functional and dysfunctional brain activities in epileptic patients. In this study, we compared SFS and SFFS strategies in order to figure out if SFS already provides near-optimal results. SFS and SFFS strategies were performed using the “sequential” function in MATLAB and sequential floating feature selection toolbox^3^, respectively.

The second component in the feature selection strategy is an objective (criterion) function to evaluate over all possible feature subsets. In this work, the misclassification rate of the linear SVM classifier was set as an objective function (Guyon and Elisseeff, 2003). The combination of SFFS and SVMs has previously been used, for example to assess Gabor features for classification of Parkinson’s disease risk assessment based on transcranial sonography images (Al-Zubaidi et al., 2013). To evaluate the feature subset, the data were divided into test and training samples using a LOOCV scheme. Accordingly, independent samples were used for training and testing. For each LOOCV loop, the training samples were submitted to train an SVM model, and the test sample was applied to that model to evaluate the feature subset. In the end, the average of the values returned by LOOCV loops was calculated and used to assess each candidate’s feature subset (Kohavi and John, 1997).

The classification accuracy (CA) was derived using a LOOCV strategy with confusion matrix (CM) and calculation of classification error rate (ER). In our study, the CM comprises information about the actual and predicted classifications generated by linear SVM. By comparing the results of the SVM classifier (hunger or satiety) with the reference data, we documented the outcomes of the CM in the present study as given in Table 2. For significance testing (Pereira et al., 2009), we estimated the empirical distribution by calculating the error rate 10,000 times for random label permutations in a cross-validation procedure. *P* < 0.05 implies that classification results differ significantly from chance.

**Table 2:** Confusion matrix.

		Reference data	
	
		Hunger	Satiety
Classified data	Hunger	$\left[\begin{array}{cc} \text{TP} & \text{FP}\\ \text{FN} & \text{TN} \end{array}\right]$	
	Satiety		


*True positive (*TP*): The number of participants that were correctly classified in hunger condition. False positive (*FP*): The number of participants that were incorrectly classified in hunger condition. False negative (*FN*): The number of participants that were incorrectly classified in satiety condition. True negative (*TN*): The number of participants that were correctly classified in satiety condition.*

## Results

**FIGURE 3:**
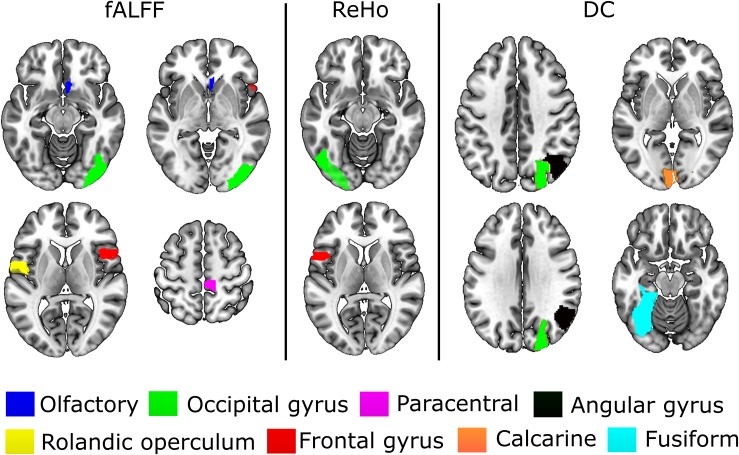
Brain regions that provided relevant information to distinguish between hunger and satiety states in healthy lean participants. The performance of these regions was evaluated by linear SVM classifier and SFFS algorithm. All images are in neurological orientation, i.e., right = right and left = left.

**FIGURE 4:**
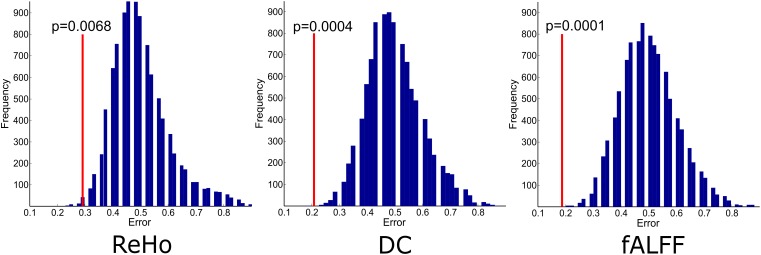
Empirical distributions of incorrect classification generated via 10000 times of random label permutations for region sets selected by SFFS. Red line shows the actual classification error.

The experiments showed that fALFF was marginally better than ReHo and DC in distinguishing between hunger and satiety states in the healthy brain (Table 3). The region subset obtained by SFFS resulted in higher classification accuracy than SFS, both higher than no feature selection (90 regions). Using a linear SVM classifier with an LOOCV strategy, we observed that the fALFF region subset selected by SFFS identified the hunger state with the highest classification accuracy of 81% and with the most balanced overall performance. In our SFFS results (Table 3), the regions 45 and 46 are the left and right inferior occipital lobe (Table 1), respectively, and region 50 is the right superior occipital lobe. Also, regions 5 and 35 are medial (Calcarine) and inferior (Fusiform) surfaces of the occipital lobe, respectively. Furthermore, regions 17 and 18 are left and right frontal gyrus, respectively, region 52 is the right Olfactory cortex, region 56 is the right Paracentral lobule and region 73 is the left Rolandic operculum. SFFS-identified brain regions, which distinguished best between hunger and satiety for each rs-fMRI feature, are shown in Figure 3. Furthermore, SFFS fALFF (Figure 4) was most stable in the permutation test (ER = 0.19 / *p* = 0.0001) closely followed by DC (ER = 0.21/*p* = 0.0004) and ReHo (ER = 0.29/*p* = 0.0068).

**Table 3:** Classification accuracy of rs-fMRI data using different models of brain connectivity/activity and features selection algorithms with linear SVM classifier.

Rs-fMRI features	90 regions				Region sets by SFS					Regions sets by SFFS				
			
	CA	CM	Sen	Spe	*R*[1, ..., 90]	CA	CM	Sen	Spe	*R*[1, ..., 90]	CA	CM	Sen	Spe
ReHo	50%	$\left[\begin{array}{cc} \text{12} & \text{12}\\ \text{12} & \text{12} \end{array}\right]$	50%	50%	*R*[22,61]	69%	$\left[\begin{array}{cc} \text{17} & \text{8}\\ \text{7} & \text{16} \end{array}\right]$	71%	67%	*R*[45,17]	71%	$\left[\begin{array}{cc} \text{20} & \text{10}\\ \text{4} & \text{14} \end{array}\right]$	83%	58%
DC	54%	$\left[\begin{array}{cc} \text{16} & \text{14}\\ \text{8} & \text{10} \end{array}\right]$	67%	42%	*R*[50,55,4,68]	71%	$\left[\begin{array}{cc} \text{19} & \text{9}\\ \text{5} & \text{15} \end{array}\right]$	79%	63%	*R*[50,4,5,35]	79%	$\left[\begin{array}{cc} \text{22} & \text{8}\\ \text{2} & \text{16} \end{array}\right]$	92%	67%
fALFF	58%	$\left[\begin{array}{cc} \text{16} & \text{14}\\ \text{8} & \text{12} \end{array}\right]$	67%	50%	*R*[61,77,35,6,1]	73%	$\left[\begin{array}{cc} \text{17} & \text{6}\\ \text{7} & \text{18} \end{array}\right]$	71%	75%	*R*[56,73,46,52,18]	81%	$\left[\begin{array}{cc} \text{19} & \text{4}\\ \text{5} & \text{20} \end{array}\right]$	79%	83%


*SFS, sequential forward selection; SFFS, sequential forward floating selection; CA, classification accuracy; CM, confusion matrix; Sen, sensitivity; Spe, specificity. Sen: Probability of a test to correctly classify a participant in hunger condition (TP) within all participants actually in hunger condition (TP + FN). Spe: Probability of a test to correctly classify a participant in satiety condition (TN) within all participants actually in satiety condition (FP + TN).*

## Discussion

The primary goal of the present research was to assess the ability to classify different brain states by applying a MVPA approach, i.e., feature selection strategies and linear SVM, on various features, i.e., connectivity parameters, derived from rs-fMRI data. This approach was carried out on a data set comprising two conditions (hungry and satiated) in a repeated measures design. As the two metabolic states, i.e., hunger and satiety, were induced for a rather long time (36 h), experimental conditions can be treated as the ground truth to compare and evaluate the classification scheme.

An advanced preprocessing, including ICA-AROMA, was carefully applied, ensuring the removal of motion artifacts and other structured noise from the data [e.g., cardiac pulsation artifacts (Pruim et al., 2015b)]. Thus, classification in the current case is deemed to reflect true brain differences rather than extracerebral differences (e.g., motion) between the conditions.

A critical question with regard to classification is the selection of the best approach for feature selection. The inclusion of all possible features and the computation of all possible combinations of features is computationally not feasible at present. Therefore, sequential search techniques have gained some popularity. These work by choosing the best individual feature and then adding a second feature that yields the best classification accuracy in combination with the first feature. This procedure is repeated for a third and any subsequent features until the addition of further features does not yield an improvement of classification rates. Alternatively, the user can predetermine the maximum number of features. The SFFS method tries to optimize feature selection by adding an elimination step to this sequential search process. Concretely, at each level, it is examined whether the classification rates improve, if any of the selected features are eliminated. If this happens, the feature set is reduced by this feature. Then, the search continues based on the new set. Jain and Zongker (1997), who compared 14 different methods for feature selection applying them to the problem of handwriting recognition. In this case, the SFFS method outperformed other feature selection schemes. It has to be pointed out, however, that conventional feature selection approaches, including SFS and SFFS as well as filter approaches (Kira and Rendell, 1992; Almuallim and Dietterich, 1994), have recently been supplemented by metaheuristic methods for feature selection. In this regard, Zhu et al. (2007) have suggested a genetic algorithm combined with local search in a hybrid wrapper and filter feature selection algorithms. Others, like Neshatian and Zhang (2009) and Gu et al. (2018), proposed new optimizations methods including such advanced feature selection procedures. Applying genetic algorithms and new optimization functions on rs-fMRI data are beyond the scope of this study and will be considered in future work.

In the present study, the subset of regions obtained by the SFFS algorithm provided the highest classification rate for all rs-fMRI maps (Table 3). Using fALFF, SFFS and SVM classification, we were able to demonstrate that patterns of amplitude BOLD signals in five brain regions [paracentral lobule, Rolandic operculum, olfactory cortex, lateral occipital (inferior division) gyrus, and inferior frontal (opercular) gyrus; Figure 3] can distinguish between metabolic states (hunger vs. satiety) with 81% accuracy.

The Rolandic operculum, which belongs to somatosensory regions, is activated during the anticipation and consumption of food (Stice et al., 2008; Stice and Yokum, 2016), in response to palatable food receipt (Stice et al., 2013) and has been associated with the processing of high- and low-caloric food pictures (Stice et al., 2010). Among many functions, paracentral gyrus is known to respond to highly rewarding stimuli (Stice and Yokum, 2016). A study using independent component analysis to estimate functional connectivity (FC) parameters showed that the connectivity strength of the paracentral gyrus in the sensorimotor network was increased during hypoglycemia relative to euglycemia (Bolo et al., 2015). Furthermore, Van Duinkerken et al. (2012) reported that the change of sensorimotor FC was associated with basal glycemic levels in type 1 diabetes mellitus patients. Thus, the paracentral lobule seems to be part of the reward system and the sensorimotor network. The olfactory cortex (OLFC), whose activity was modulated by metabolic states as well, is involved in the experience and processing of negative affective states, including anxiety and depression (Krusemark et al., 2013). Consistent with that, a rs-fMRI study in rodents demonstrated that ReHo of the OLFC is increased in stress-exposed rats compared to a control group (Li et al., 2018). In our study, the fasting for 36 h might have led to a stress increase, which might be reflected in an increase in OLFC activity. Moreover, the satiety state might have reduced peripheral hunger signals compared to the hunger state, and accordingly, might have influenced brain regions related to somatosensory processes, such as Rolandic operculum, and parts of the sensorimotor network like the paracentral lobule.

The inferior frontal gyrus (IFG) has been suggested to be involved in cognitive control (Hare et al., 2009; Sundermann and Pfleiderer, 2012). IFG activation during response inhibition has been associated with a reduced desire for food and with successful impulse regulation (Gabrieli et al., 2015; Hollmann et al., 2012; Lopez et al., 2014). In addition, stronger IFG activity in response to orosensory stimulation was found in successful weight loss maintainers compared to people who were obese or normal weighted (Sweet et al., 2012). In our experiment, participants had to refrain from eating during the hunger state and from overfeeding during the satiety state, which may have contributed to the finding that the IFG is partially important for classifying between different metabolic states.

The lateral occipital cortex (LOC) is part of the visual association cortex and is activated in response to the perception of emotionally salient stimuli, such as food, which is thought to be a correlate of heightened attention (Killgore and Yurgelun-Todd, 2007; van der Laan et al., 2011). For instance, a recent rs-fMRI study using SVM on graph theory analysis indicates that the LOC is partly important for classification between high-caloric (potato chips) vs. low-caloric (zucchini) food ingestion on the brain of healthy subjects (Mendez-Torrijos et al., 2018). Furthermore, it has been suggested that the processing of visual salience of a stimulus depends on the affective state of the individual and the motivational value of a stimulus (Killgore and Yurgelun-Todd, 2007). Considering the general role of the LOC in the visual processing of food stimuli, this region might potentially facilitate the detection/perception of such cues in a deprived state. Note that these interpretations are based on reverse inference of resting-state data and should thus be taken with caution.

However, some studies have used rs-fMRI to investigate changes in baseline brain activity of lean or obese participants during both hunger and satiety states. For instance, Lohmann et al. (2010) showed increased centrality, which was measured by eigenvector centrality analysis, of the anterior precuneus (APCUN) during the hunger state relative to the satiety state of 22 normal volunteers. Consistent with that, our previous study (Al-Zubaidi et al., 2018) revealed that the fALFF was increased in the APCUN and posterior cingulate cortex (PCC) when comparing hunger against satiety states. Additionally, Wright et al. (2016) used seed-based connectivity analysis to estimate the FC parameters of 19 healthy participants. They reported that the FC between the posterior insula and superior frontal gyrus, and between the hypothalamus and IFG, were enhanced during the hunger state. Furthermore, it has been found that both 20 lean and 20 obese subjects had increased ReHo connectivity from hunger to the satiety state in the orbitofrontal cortex and inferior temporal lobe (Zhang et al., 2015). These studies reported different brain regions that might be related to the changes in the metabolic state compared to the results of the current study, except IFG. These inconsistencies here might be associated with the different experimental paradigms and neuroimaging modalities.

Importantly, the statistical analysis at group levels of the studies mentioned above was computed by using GLM approaches to define the significant brain regions at baseline activity that are sensitive to changes in metabolic state. In GLM approaches, the *p*-values are the successful statistical tests to represent significant brain regions that show different brain activities in the average sense of one or more brain features when compared between different groups and/or experimental conditions. On the other hand, SVM classifier aims to automatically classify each subject into one of the groups or experimental design in the study. Thus, the overall classification accuracy is usually used to measure the success of the studies. In general, it is easier to demonstrate group or experimental condition differences compared to predict a single subject (Arbabshirani et al., 2017). Furthermore, the significant variables or features that show the difference between group or experiment do not necessarily have high classification accuracy and vice versa (Lo et al., 2015; Arbabshirani et al., 2017). Hence, brain regions that showed significant differences between hunger and satiety states in previous studies do not necessarily mean that these regions can predict the subject class with high accuracy and vice versa.

Furthermore, our results indicate that fALFF analysis is more informative than ReHo and is slightly more precise than DC for classification of resting brain changes during hunger and satiety, probably because fALFF is an index of the power of the BOLD signal. Against this, ReHo and DC parameters refer to dynamics of BOLD connectivity, either with some (in this case, 26) neighboring voxels (ReHo) or with all voxels (DC) in the gray matter of the brain.

One other important question is, whether SFFS based classification is superior to simply trying to classify states using statistically significant group differences. Baker et al. (2012) answered this question on an EEG data set from AD patients, MCI patients and HC. They used an SFFS algorithm and a *t*-test to classify patients and found that the SFFS technique resulted in improved classification rates compared to the *t*-test for four feature types (average power, coherence, correlation, and phase synchrony). They concluded that the SFFS algorithm selects reliable features for classification where statistically significant features fail in classification.

The sample size of most fMRI studies is often relatively small due to the high costs of scanning time and subject stamina (Poldrack et al., 2017). However, sample size impacts the trade-off between accuracy and generalizability (Schnack and Kahn, 2016). For instance, in the context of rs-fMRI features and SVM classification methods, several studies reported high classification accuracies (92 ± 9%) with relatively small sample sizes (20 ± 5 subjects per group), when classifying groups of brain disease patients and healthy subjects (Lord et al., 2012; Tang et al., 2012; Fekete et al., 2013; Wang et al., 2013; Wei et al., 2013; Cheng et al., 2015; Khazaee et al., 2015b). Here, the high classification accuracy is driven by the heterogeneity between groups (Schnack and Kahn, 2016; Arbabshirani et al., 2017). In contrast, studies with large sample sizes are assumed to result in classification models with a higher degree of generalizability, allowing for a better prediction in samples drawn from other populations. Their classification models capture a more complete picture of disease patterns, but at the cost of lower accuracy, which is likely due to the within-group heterogeneity (Anderson and Cohen, 2013; Krystal et al., 2013; Guo et al., 2014; Schnack and Kahn, 2016; Arbabshirani et al., 2017).

One limitation of the present study is the ability to generalize, since the sample size of 24 subjects is relatively small. Accordingly, larger samples are needed to confirm our findings. However, we would like to argue that our results are not driven by the heterogeneity between samples, because we have chosen a within-subject design in a well-controlled experimental setting. Also, we evaluated whether the rs-fMRI features in conjunction with sequential feature selection strategies were sufficiently reliable to predict the subject’s metabolic state using the LOOCV scheme. Thus, independent training and testing samples were used to estimate the SVM model parameters and to validate the classification model. In this case, the CA was derived by averaging the resulting classification accuracies over all samples. All in all, the homogeneity of our samples and the high CA results in an increased validity of our findings, determining brain patterns that are able to discriminate between different metabolic states.

As rs-fMRI has received widespread attention over the past 10 years, the possibility of reliable classification of disease conditions or subject states (e.g., sleep stages) paves the way for using rs-fMRI as a diagnostic tool on an individual patient/subject level. In addition the applications mentioned in the introduction, such as the prediction of conversion of MCI to AD (Hojjati et al., 2017), many other diagnostic and research questions lend themselves to this approach, e.g., the differentiation of typical Parkinson’s disease from atypical Parkinsonian syndromes (c.f., Tang et al., 2010). From our data, we conclude that fALFF, in combination with SFFS based feature selection, is a useful and straightforward way to proceed in tackling such research questions.

## Ethics Statement

This study was carried out in accordance with the recommendations of the Declaration of Helsinki. The protocol was approved by the ethics committee of the University of Lübeck. Before participation, each participant gave written informed consent to the study.

## Author Contributions

KJ-C and TM designed the study and wrote the protocol. AA-Z, MH, and KJ-C participated in the data collection. AA-Z managed the literature searches, wrote the first draft of the manuscript and performed data analysis. AA-Z and AM designed the statistical analyses. MH and TM helped with interpretation of data. All authors contributed to the manuscript revision and approved the final version.

## Conflict of Interest Statement

The authors declare that the research was conducted in the absence of any commercial or financial relationships that could be construed as a potential conflict of interest.

## Footnotes

1 http://fsl.fmrib.ox.ac.uk/fsl/

2 http://www.alivelearn.net/xjview8

3 http://splab.cz/en/download/software/software-pro-sekvencni-selekci-priznaku

## Abbreviations

AAL: Automated-Anatomical-Labeling
AD: Alzheimer’s disease
APCUN: anterior precuneus
BMI: body mass index
BOLD: Blood-oxygen-level-dependent
CA: classification accuracy
CFS: cerebrospinal fluid
CM: confusion matrix
DARTEL: diffeomorphic anatomical registration through exponentiated Lie algebra
DC: degree of centrality
DPARSFA: data processing assistant for resting-state fMRI advanced edition
EPI: echo-planar imaging
ER: error rate
fALFF: fractional amplitude of low-frequency fluctuations
FC: function connectivity
fMRI: functional magnetic resonance imaging
FSL: FMRIB Software Library
GLM: general linear model
HC: healthy controls
ICA-AROMA: independent component analysis (ICA)-based strategy for automatic removal of motion artifacts
IFG: inferior frontal gyrus
KCC: Kendall’s coefficient concordance
LOC: lateral occipital cortex
LOOCV: h-out cross-validation
MCI: mild cognitive impairment
MLC: machine learning classifier
MNI: Montreal Neurological Institute
MVPA: multivariate voxel-pattern analysis
OLFC: olfactory cortex
ReHo: regional homogeneity
ROI: region of interest
rs-fMRI: resting-state functional magnetic resonance imaging
SFFS: sequential forward floating selection
SFS: sequential forward selection
SPM: statistical parametric mapping
SVM: support vector machine
TE: echo time
TR: repetition time
